# Comparative Study on the Efficacy and Safety of Tumor Resection in Vitrectomy for Retinal Vasoproliferative Tumors

**DOI:** 10.1155/2019/7464123

**Published:** 2019-01-02

**Authors:** Bin Zheng, Yan Chen, Lifeng Chen, Huan Chen, Jingwei Zheng, Feng Chen, Zongming Song, Lin Fu, Xuting Hu, Jiandong Pan, Hengli Lian, Lijun Shen, Qiuming Li

**Affiliations:** ^1^The First Affiliated Hospital of Zhengzhou University, Zhengzhou, Henan 450052, China; ^2^Eye Hospital, Wenzhou Medical University, Wenzhou, Zhejiang 325000, China; ^3^Henan Eye Hospital, Henan Eye Institute, Henan Provincial People's Hospital and People's Hospital of Zhengzhou University, Zhengzhou, Henan 450052, China

## Abstract

**Purpose:**

To investigate the efficacy and safety of combined vitrectomy with tumor resection in the treatment of retinal vasoproliferative tumors (RVPT).

**Methods:**

Retrospective study. RVPT patients who underwent vitreous surgery at the Eye Hospital of Wenzhou Medical University from January 2011 to July 2017 were included. The main outcomes included treatment type, tumor activity, and best-corrected visual acuity (BCVA).

**Results:**

Altogether, 16 patients with 17 eyes were enrolled with follow-up of no less than 6 months. Eight eyes were in the resection treatment group (Group R) and 9 eyes were in the conservative treatment group (Group C). Female (69%) were more common. The mean age was 50 (49.72 ± 12.92) years. Fifteen patients got unilateral onset and only one patient suffered bilaterally. The common symptoms were decreased visual acuity, floaters, and visual distortion. The preoperative BCVA ranged from hand movement to 20/20, with an average of 0.82 ± 0.75 LogMAR. Patients were all not high myopia, with a mean axial length of 23.27 ± 0.27 mm (21.61 mm to 24.67 mm). Of the retinal diseases, the epiretinal membrane was the most common, followed by vitreous hemorrhage, uveitis, subretinal fluid, and so on. Compared with the baseline BCVA, it improved more at postoperative 6 months and the last visit in Group R than in Group C (*P*=0.006 and *P*=0.033). The BCVA-improved 0.2 LogMAR or above in 6 months was 2 eyes in Group C and 7 eyes in Group R. All tumors in Group R were completely resected, whereas three in Group C (33.3%) had definite activity (*P*=0.008). In all samples, tumors were located on the inner side of the retina and had small vessel wall thickening and hyaline degeneration. The degree of astrocyte proliferation varies widely among different tumors.

**Conclusions:**

RVPT was more likely to occur in nonhigh myopia patients. Epiretinal membrane and vitreous hemorrhage were the main causes for vitreous surgery in RVPT patients. Compared with conservative treatment, surgical resection of the tumor is more beneficial to patients on visual acuity recovery and preventing tumor relapse. It is a safe and effective way to treat RVPT.

## 1. Introduction

Retinal vasoproliferative tumors (RVPT) are rare retinal-derived benign and acquired vascular tumors and occur frequently in healthy adults [1–4]. Currently, treatment modalities for RVPT are generally divided into conservative treatment (nonresection) and surgical resection. Conservative managements are the most commonly used methods for RVPT, including cryotherapy [5–7], laser photocoagulation [6, 8], radiotherapy [9, 10], anti-VEGF treatment [11], and photodynamic therapy [12, 13]. These are more effective on small tumors, but patients with concomitant complications and larger tumors may require multiple treatments. Traditionally, vitreous surgery was mostly aimed at the treatment of RVPT-related intraocular complications, such as macular epiretinal membrane, vitreous hemorrhage, and traction retinal detachment. During operation, the tumor was not removed, and only conservative treatments such as cryotherapy and laser photocoagulation are performed [6, 8, 14–16]. However, more than 30% of the tumors were not effectively controlled and turned to tumor resection in vitrectomy (Table 1) [6].

In this study, the safety and efficacy of intraoperative tumor resection in vitrectomy of RVPT were compared with the long-term results of tumor conservative managements in vitreous surgery.

## 2. Patients and Methods

This is a retrospective study. RVPT patients who underwent vitreous surgery at the Eye Hospital of Wenzhou Medical University, Zhejiang, China, from January 2011 to July 2017 were included. Altogether, 16 patients with 18 eyes were included, accounting for 1.20‰ of the total surgical number at the same period.

### 2.1. Data Collection

Preoperative data were collected from all patients for analysis of entire clinical features. Baseline characteristics included gender, age, systemic disease, eye types, major symptoms, duration of symptoms, best-corrected visual acuity (BCVA), axial length, spherical equivalent, intraocular pressure, and concomitant retinal diseases. The main records of the tumor consisted of tumor type, number, location, size, color, whether it was found before surgery, and whether it could be detected by B-ultrasound.

Intraoperative complications were recorded. Postoperatively, follow-up visits, complications at 1 month, BCVA at 6 months and the last follow-up, associated retinal diseases, retreatment method, and tumor activity were all collected.

The BCVA results were converted to LogMAR vision record. The criteria for the tumor activity were as follows: (1) fluorescence angiography showed that there was leakage of fluorescence at the tumor site after more than 3 months follow-up and (2) there was subretinal fluid effusion or exudation connected to the tumor body and was not resolved or even progressed after at least 3 months follow-up. The tumor was determined to be active when one of the criteria is met.

### 2.2. Groups

**Figure 1:**
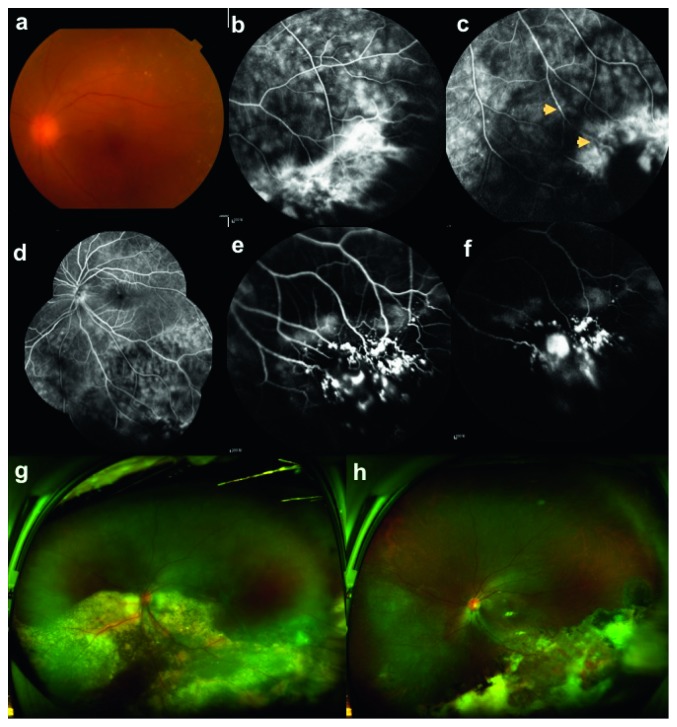
Case 6, female, 42 years old, decreased vision in the left eye with 1 week of shadow flutters before eye, best-corrected visual acuity 0.5, vitreous bloody opacity, cells (++), blurred fundus, and 2PD pink tumor in the inferior temporal area. (a–c) Before the first surgery: (a) color photo of the fundus; (b) fluorescein angiography at 2 minutes and 15 seconds, a mottled hyperfluorescent zone was seen in the inferior temporal location, where the vessels dilated like tumor with obvious surrounding capillary leakage; (c) fluorescein angiography at 2 minutes and 28 seconds, where some blood vessels entered the hyperfluorescence phase with uneven diameters and slightly expanded (flat arrows). (d) FA angiography at 2 minutes and 46 seconds shows a clear boundary between the subretinal effusion and the normal retina, and the common leakage of capillaries. (e) ICG angiography at 1 minute and 16 seconds shows that the blood vessels in the lesion expand and distort, mostly in coarse granules, clustered into clusters, and the plaque-like fluorescence filling in the tumor. (f) ICG angiography at 10 minutes and 14 seconds. At this time, the intensity of intravascular fluorescence decreased and part of the tumor area also reduced, resulting in a high fluorophore mass. She was injected anti-VEGF once and triamcinolone acetonide twice, while her condition could not be controlled. The BCVA before the second surgery was 0.05. (g) Optomap illustrated retinal detachment and hard exudation involving the posterior pole. No conservative treatment was performed after surgery, and BCVA at 30 months after surgery was 0.1. (h) the Opel image showed that the pigment epithelium of the original detached area was atrophic, scarring, and affected by the macular center.

Based on whether the tumor was removed during surgery, the patients were divided into conservative treatment group (Group C) and resection treatment group (Group R). The follow-up period was not less than 6 months. In one case (case 5), the follow-up period was shorter than 6 months and was excluded. In case 6, the patient underwent surgery twice. The first nonresection surgery was included in Group C in which a total of 9 eyes were involved. The second resection operation was recruited in Group R (Figure 1) for a total of 8 eyes. All patients had signed informed consent for surgery before and after treatment.

### 2.3. Surgery

The surgery was performed by 3 surgeons in our hospital (Dr. BZ, Dr. FC, and Dr. ZS). After the retrobulbar anesthesia, 23G or 25G vitrectomy was performed. Intraoperative management to the lens was based on the three principles: (1) if the lens opacities seriously affected the operator, the lens was removed and the intraocular lens was implanted at the same time; (2) if silicone oil was applied, the lens was removed in a second surgery; and (3) if silicone oil was not used and the patient was older than 50 years, the lens was also removed and the intraocular lens was implanted. The lens was kept if the patient was less than 50 years old.

After removal of the central vitreous body, the vitreous cortex around the optic disc was separated. Some of the patients were assisted with triamcinolone acetonide. The epimacular membrane was stripped and stained with indocyanine green while removing the inner limiting membrane, approximately 4 PD in diameter. Group C only used cryotherapy or laser photocoagulation treatment on the tumor body. The tumor body was pale by cryotherapy, and the spot covered the tumor body by laser photocoagulation. Some patients performed electrocoagulation on the tumor's nourishing and draining blood vessels. Patients in Group R were treated with prophylactic photocoagulation around the tumor, and the areas with abnormal retinal vessels were covered. A retinectomy was conducted close to the tumor body, and the tumor was dissociated. Some tumors were removed from a scleral incision 4 mm posterior to the limbus. After gas-liquid exchange, the vitreous was filled with BSS, long-acting gas, sterilized air, or silicone oil. Some of them received intraocular injection of triamcinolone acetonide 0.1 ml (4 mg). In 3 to 6 months after surgery, all silicone oil was removed from silicon oil tamponade eyes.

### 2.4. Pathological Examination

Tumor samples from 5 patients with RVPT (cases 12, 13, 14, 15, and 16) in the resection Group R were routinely sent for HE staining for pathological analysis.

### 2.5. Statistical Analysis

This study used SPSS 23.0 software for statistical analysis. Quantitative data were evaluated by the Kolmogorov–Smirnov (K-S) test for the normality. The normal distributed data were compared by *t*-test or repeated variant analysis, and the results were presented as the mean ± SD (standard deviation of the mean). Categorical variables were assessed using the chi-square test or Fisher's exact test. Statistical significance was set at *P* value < 0.05.

## 3. Results

### 3.1. Overall Data

In this study, 16 patients with 18 eyes were enrolled (Table 2). In total, female consisted of 69%. The mean age was 50 (49.72 ± 12.92) years, with the youngest at 28 years old, the oldest at 69 years old, and 81% of them older than 40 years. Of those patients, 4 (25%) presented with hypertension, 1 (6%) immune system disease, 1 (6%) pulmonary tuberculosis, and 1 (6%) endometrial carcinoma. The incidence of the left eye (59%) is slightly higher than that of the right eye (41%). Fifteen people got unilateral onset and only one patient suffered bilaterally. The patient's clinical symptoms were more common with visual loss (89%), followed by floaters (39%), visual distortion (19%), visual field defect (6%), photopsia (6%), and red eye (6%). The mean duration of symptoms was 7.88 ± 10.86 months, with the shortest one being 0.25 months and the longest being 36 months. The averaged preoperative BCVA was 0.82 ± 0.75 LogMAR ranging from hands moving before eye to 20/20. The preoperative IOP values were all normal. No patient got high myopia, and the axial length ranged from 21.61 mm to 24.67 mm, with the mean value of 23.27 ± 0.27 mm. The primary RVPT was 11 (65%) eyes, and the secondary RVPT was 6 (35%) eyes. Both were associated with uveitis. The retinal disease was most common with the epimacular membrane (67%), followed by vitreous hemorrhage (39%, 7 eyes, including 4 eyes 1 grade, 1 eye 3 grade, and 2 eyes 4 grade), uveitis (39%), subretinal fluid (28%), retinal exudate (23%), traction retinal detachment (18%), and macular holes (6%).

**Figure 2:**
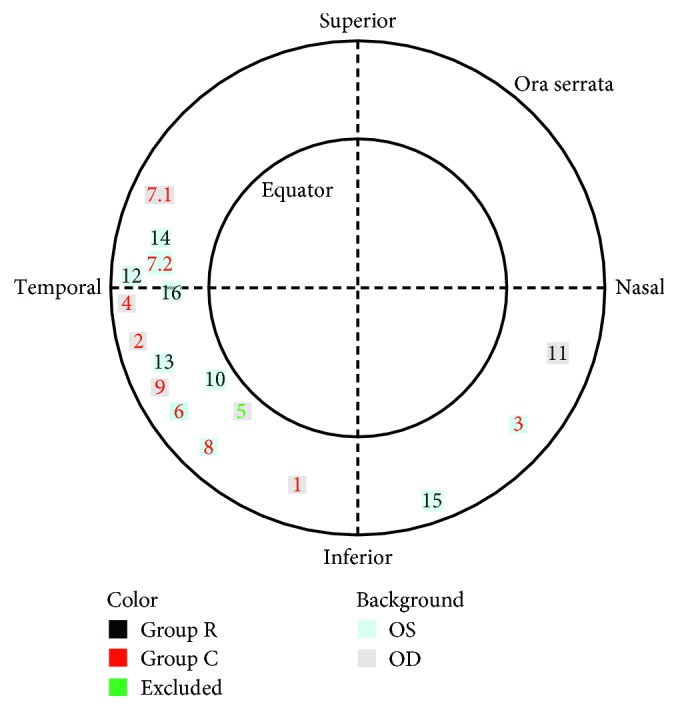
Schematic demonstration of tumor location.

Preoperative physical examination revealed tumor in 12 eyes, 3 of which were also detected by ultrasound. All tumors were isolated, focal lumps. The tumor located commonly inferiorly (70%) and temporally (72%), of which 53% was inferior temporal, followed by the superior temporal (23%) and the inferior nasal (18%) location. There was no lesion at the superior nasal area (Figure 2). The tumor size was mostly concentrated in 2-4PD size (82%).

### 3.2. Intergroup Data

For the baseline characteristics, only gender was significantly different between these two groups (*P*=0.029). The eye, age, duration of symptoms, preoperative BCVA, follow-up time, axial length, spherical equivalent, and intraocular pressure all showed no differences (Table 3).

Both at the 6 months after surgery and last follow-up visit, the BCVA was not statistically different between Group C (0.52 ± 0.64 and 0.47 ± 0.56) and Group R (0.33 ± 0.31 and 0.43 ± 0.31). For the improvement of BCVA at 6 months after surgery and the last follow-up visit compared with the baseline level, both were significantly different between Group C (0.03 ± 0.43 and −0.03 ± 0.48) and Group R (−0.86 ± 0.70 and −0.76 ± 0.73) (*P *= 0.006 and *P*=0.033) (Table 3). For the improvement of BCVA at least 0.2 LogMAR at 6 months after surgery and the last follow-up visit, there were 2 eyes (22.2%) in Group C and 7 eyes (87.5%) in Group R. Two eyes (22.2%) in Group C presented with vision loss. One was case 6.1, and the tumor was active after surgery, resulting in subretinal fluid with macular involvement (Figure 1). The other one was case 7.1, and the tractional epiretinal membrane secondary to the postoperative survival tumor along the superior temporal retinal vasculature arch involved the macula. One eye (12.5%) in Group R got vision reduction. It was case 12 which was induced by the posterior capsular opacity and the clumpy silicone oil particles adhered to the posterior capsule center. For the BCVA that decreased at the last follow-up compared with 6 months after surgery, there was one eye in Group C. It was case 1 caused by the complicated posterior capsular opacity. And there were 4 eyes in Group R. They were case 11, case 12, case 13, and case 16. The former two were caused by after cataract and the latter two developed central posterior capsule opacity.

**Figure 3:**
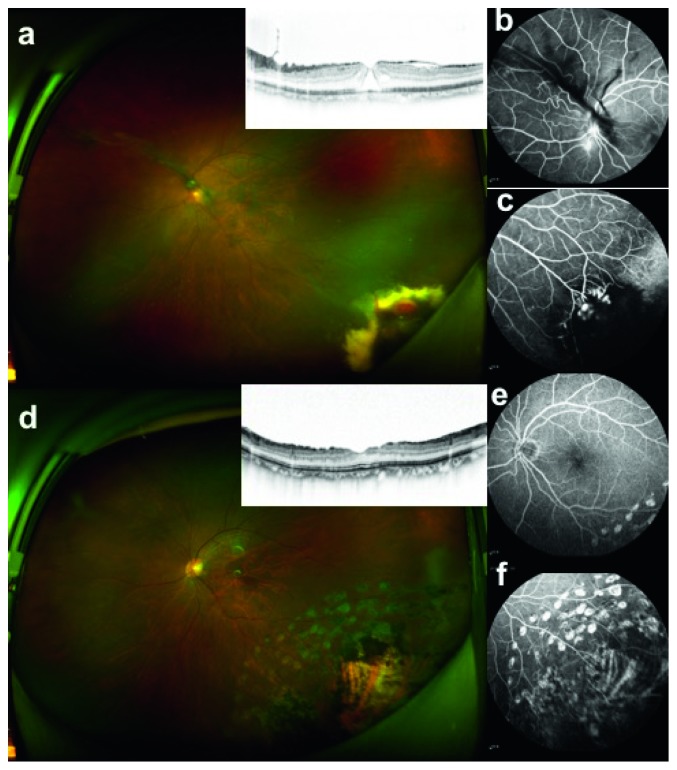
Case 8, female, 60 years old, left eye blurred for more than 3 years and 1 month, BCVA was 0.6. (a–c) Before surgery: (a) optomap showed an approximately 4 PD pink tumor-like mass around the peripheral retina at 5 o'clock, surrounded by a large number of yellow-white hard exudation and local tractional retinal detachment on the superior temporal area; illustrated OCT showed a macular hole. (b) FA imaging at 12 minutes and 19 seconds. The para-optic disc area showed hyperfluorescence with nasal vessels distorted. (c) FA imaging at 1 minute and 17 seconds showed that the blood vessels in the tumor bulged and expanded in clusters; the fluorescence filling degree was staggered, the surrounding fluorescence was blocked, or the fluorescence was seen through the sheet. Combined anterior and posterior segments surgery was conducted with cryotherapy, stripping the inner limiting membrane, filling with C3F8 gas, and injection of triamcinolone acetonide. She was followed up for 17 months, and the BCVA was 0.8. (d) Optomap displayed atrophic scars in the tumor and the inset demonstrated macular hole closure. (e) FA imaging at 14 minutes 48 seconds (leakage area around the optic disc disappeared). (f) FA imaging at 13 minutes 54 seconds. The tumor atrophied and disappeared, surrounded with scattered laser spots without fluorescence leakage.

In terms of tumor activity, all the 8 eyes in Group R were completely removed. Six eyes (66.7%) in Group C were completely inactivated (Figure 3), whereas 3 eyes (33.3%) were still active, including case 4, case 6.1, and case 7.1, in which case 4 was treated by laser therapy, and in case 6.1, the tumor was surgically removed thereafter (Figure 1). There was a statistically significant difference between the two groups (*P*=0.008) (Table 4).

The complications within 1 month after the operation included mainly increased IOP and hemorrhage, which were improved by medication. In case 11 of Group R, the foot loop of the artificial lens prolapsed into the anterior chamber and was re-surgically reset.

**Figure 4:**
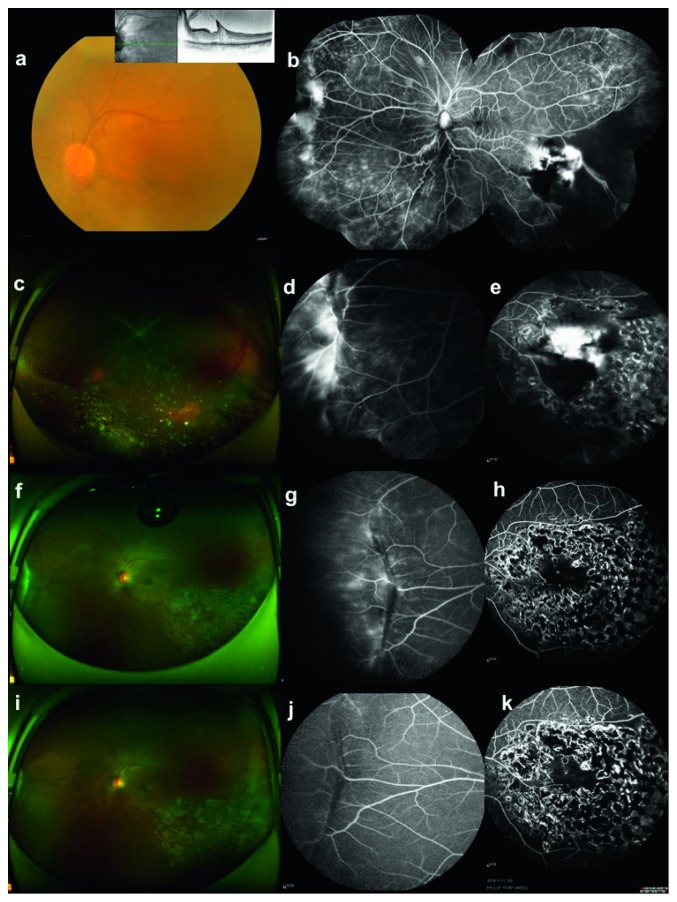
Case 10, male, 46 years old, left eye blurred for more than 5 months with micropsia and metamorphopsia for more than 2 months, a history of ankylosing spondylitis for 8 years, the left eye BCVA was 0.4. (a–c) First visit examination: (a) turbid optical media, astral vitreous degeneration, visible neovascular membrane in front of disc, and illustration OCT showing traction in the macula center. (b) FA imaging at 1 minute and 18 seconds. Inferior temporal part of the tumor leaked obviously, and the nasal retinal leaked. The peripheral retinal was observed with diffuse capillary leakage. Photocoagulation therapy was given to the tumors. BCVA after 10 months follow-up was 0.2. (c) Optomap revealed a 2PD pink tumor on the inferior temporal position, surrounded by standing laser spots. (d) FA angiography at 2 minutes and 17 seconds. The blood vessels in the ridge-like bulge region were still leaking on the nasal side, and the surrounding capillaries leaked. (e) FA imaging at 5 minutes and 36 seconds. The original nonperfusion area scattered in the laser spot; the tumor was still significantly leaking. After 5 months, the visual acuity of the left eye suddenly decreased and was 0.02. Vitreous hemorrhage was observed and the vitreous surgery was performed with tumor resection and then filled with C3F8. After 2 months of follow-up. (f) Optomap showed that the tumor on the inferior temporal area was removed, with localized laser spots. (g) FA imaging at 10 minutes and 27 seconds. The vascular leakage in the nasal ridge-like region was significantly reduced. (h) FA imaging at 28 seconds demonstrated scattered old laser spot and no fluorescence leakage in the tumor area. After 28 months of follow-up, the BCVA of the left eye was 0.8, and the lens was slightly cloudy. (i) Optomap fundus photography: inferior temporal localized old laser spots. (j) FA imaging at 11 minutes 27 seconds. Vascular leakage in the ridge-like area disappeared. (k) FA imaging at 37 seconds. No fluorescent leakage was observed in the tumor bed.

Persistent intraocular inflammation was the most common retinal disease during the entire follow-up period. Two (22.2%) eyes in Group C and 5 eyes (62.5%) in Group R had preoperative intraocular inflammation. All patients were significantly relieved after surgery (Figure 4). The recurrent epiretinal membrane was a common long-term complication. Three eyes (33.3%) in Group C were case 4, case 7.1, and case 7.2. The former two were epimacular membrane, and the tumors were not inactivated. Case 7.2 only presented with localized epiretinal membrane proliferation around the tumor. One patient (12.5%) in Group R, case 16, for whom the inner limiting membrane was not removed during the operation showed sustained mild intraocular inflammation. In other cases, submacular exudation (case 6.1) and macular neovascularization (case 4) occurred in 2 eyes in the noninactivated tumors of Group C.

### 3.3. Pathological Results

**Figure 5:**
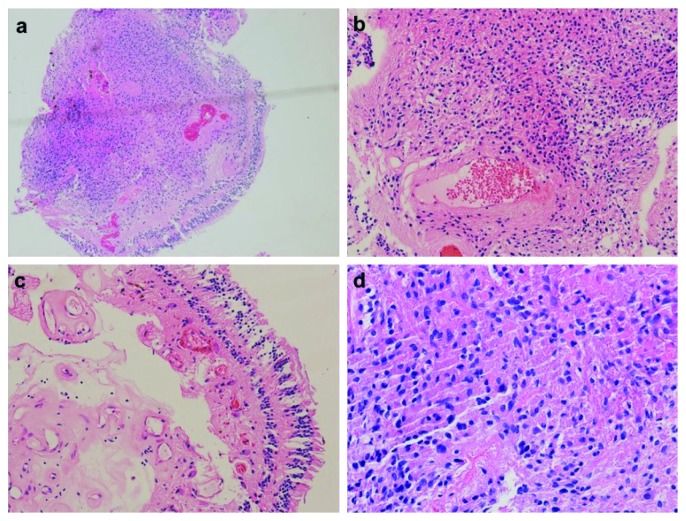
HE staining of case 14. (a) Microscopically, the tumor was involved in the inner layers of the retina, and some of the inner nucleus layer structure was broken off. Boundary between the tumor and retina was unclear (×40). (b) Glial cells arranged in a staggered weave around the hyaline degenerative vessels (×100). (c) In the inner side of retina, hyperplastic small vessels, hyaline degenerative vessel wall, and interstitial infiltration of little lymphocyte can be observed (×100). (d) High magnification showed that the proliferating glial cells were bipolar spindle cells with interlaced arrangement. The nucleus was oval or short fusiform, with fine chromatin. The nucleolus was not obvious or there were no nucleoli (×200).

In general, all samples were located on the inner side of the retina. The tumor boundary was relatively clear, and the outer surface of the retina was smooth and intact. Under the microscope, the tumor and the retinal inner layers combined tightly without obvious boundary, which involved the retinal nerve fiber layer, ganglion cell layer, and inner plexiform layer. Some individuals invaded through the inner nuclear layer, and the tumor and retina layers were interlaced, but they were all confined to the inner layers of neural retina (Figure 5(a)). In all samples, small vessel wall thickening and hyaline degeneration were observed, and fibrinous exudation and inflammatory cell infiltration were observed around some blood vessels (Figures 5(b) and 5(c)). The degree of astrocyte proliferation varies widely among different tumors. The proliferative astrocytes were interlaced or chrysanthemum-like, with the morphology of bipolar spindle-shaped cells. The nucleus was oval and short fusiform, with fine chromatin. There were no obvious nucleoli or no nucleoli, and no mitotic activity was observed (Figure 5(d)).

## 4. Discussion

RVPT is a rare retinal benign lesion. The included patients only accounted for 1.2‰ of the total retinal surgeries in our hospital during the same period. Because of its low incidence, some ophthalmologists often fail to recognize the examination and diagnosis of RVPT. The common clinical symptoms of RVPT are vision loss, floaters, visual field defect, photopsia, and visual distortion [3]. The decline of vision is often the main reason for the clinic visiting of patients. The major manifestation is gradual or sudden vision loss, which is mainly related to epimacular membrane, vitreous hemorrhage, and inflammatory opacity [1–3]. In our cases, the vision loss as the main symptom accounted for 89%. The epimacular membrane was 67%, which was much higher than the incidence of previous reports of 25%, [3] mainly because we focused on patients who received vitreous surgery. The other symptoms were followed by vitreous hemorrhage (39%) and uveitis (39%). In the course of this study, we found that missed diagnosis or misdiagnosis often occurs in some patients with mild vitreous hemorrhage. Because the intraocular mass often occurs proximal to the ora serrata. this was difficult to be detected without careful examination after pupil dilation if merely relied on B ultrasound. Of the 12 eyes being diagnosed before surgery, only 3 eyes were also diagnosed by B ultrasound. Some patients presented mainly with intraocular inflammation, often misdiagnosed as “uveitis.” They were given long-term glucocorticoid treatment until vitreous hemorrhage and referred to retinal surgery department. Therefore, for patients with vitreous hemorrhage accompanied by intraocular inflammation, doctors should be vigilant; timely pupil dilation examination for early detection and diagnosis of RVPT is very important.

Uveitis is a common cause of secondary RVPT [2, 3, 23], but the causal relationship between the two is still unclear and controversial. Some scholars believe that intraocular inflammatory cells are “spilled” from the abnormal blood vessels of the tumor [5]. It is also believed that it is the immune response to the abnormal tumors [24]. In our study, 7 cases were diagnosed as uveitis before surgery, and none of the patients treated with glucocorticoids had been relieved. Interestingly, in these patients, after removal of the tumor or inactivation of the tumor through conservative treatment, the intraocular inflammation was significantly reduced and there was no recurrence during follow-up. Therefore, we believe that intraocular inflammation may be a result of RVPT relapse.

Previous literature did not record the axial length and diopter of the patients. The axial length of our patients ranged from 21.61 mm to 24.67 mm, with an average of 23.27 mm. All patients had mild to moderate refractive errors and had no high myopia. We suppose the impaired microcirculation was caused by the thinning of choroidal thickness in high myopia [25] and the thinning of blood vessels due to the passive pulling from the retina [26]. Although VEGF may be involved in the pathological process of RVPT [19], it may be one of the reasons for the absence of RVPT in patients with high myopia due to prolonged ischemia and hypoxia that make it insensitive to increased VEGF.

According to the current reports, the thickness of the tumor based on echographic measurement is an important factor affecting the effect of conservative treatment [5, 6]. Although the application of brachytherapy has a significant effect on the healing of tumors larger than 2 mm thick, it is expensive and technically difficult for widespread use [9]. Cryotherapy is currently the most common treatment for RVPT and the most common treatment used in this study. It is effective on tumors less than 2 mm thick [5]. This is a retrospective study, with only 3 cases detected in the preoperative B-ultrasound. So the limitation of tumor thickness was not recorded and whether the thickness of the tumor affected the conservative treatment group could not be fully discussed.

For patients with complications and larger tumors, multiple treatments were required. Ineffective cases were considered to undergo a vitreous surgery to remove the tumor, and in severe cases, the eyeball was extracted [27] (Table 1). In the past, the purpose of vitreous surgery was mainly to resolve RVPT-related intraocular complications such as epimacular membrane and vitreous hemorrhage, and the tumor was not removed. In 2015, Garcia et al. reported 31 eyes in 30 patients with RVPT (17 eyes underwent vitrectomy). After the initial treatment, the recurrent rate of RVPT was 35% [6]. In our study, 9 patients in Group C were treated for this purpose. The proportion of tumors active after treatment was 33%.

Hyaline degenerative vessels are the most characteristic and repetitive pathological features of RVPT [5, 15]. Our pathological results showed varying degrees of fibrinous exudation and inflammatory cell infiltration around the vessel wall. This may be related to clinically relevant subretinal fluid, hard exudation, and even “overflow” of inflammatory cells. Therefore, we believe that the key to successful treatment is the destruction and removal of abnormal blood vessels. Now research agrees that RVPT is a benign hyperplasia without invasiveness [2, 4, 27]. Our study also found that the tumor had a clear boundary, the lesion was confined to the retinal inner layers, and there was no adhesion to the surrounding tissue. These provide a pathological basis for the feasibility of surgical resection and the choice of surgical route.

The current literature reports that the surgical removal of tumors by the vitreous approach is mainly a remedy after failure of several conservative treatments [16, 18]. There were few reported cases of direct resection while the condition of these cases was effectively controlled after this operation [16, 19, 20, 22]. In our Group R, no residue or recurrence of tumors was observed after surgery, and related intraocular complications were also cured. We believe that this is related to benign intraretinal hyperplastic tumors in RVPT itself [16, 28]. The tumors are isolated with clear borders [15], making it possible to completely remove the tumors during surgery. Moreover, similar to retinal capillary hemangiomas, RVPT also has feeding and draining blood vessels. However, these blood vessels only slightly dilate and are completely different from the remarkably thick, distorted blood vessels, as seen with the retinal capillary hemangiomas of von Hippel–Lindau disease [2]. During surgery, no special control of blood pressure is required to reduce bleeding. In 2008, Gibran reported 3 patients who were not responding to other treatments. All patients underwent intraocular tumor removal and intraocular lens implantation for cataract. Very severe fibrinous uveitis occurred in 2 eyes after surgery. Finally, the artificial lens was removed and an iris clip-type IOL was implanted. The author believed that the combination of anterior and posterior segments surgery in RVPT patients would aggravate the destruction of the blood aqueous barrier, and it was prone to induce severe anterior inflammatory reactions [16]. However, in the two groups of patients we observed, the postoperative inflammatory response was mild and no similar complications occurred. The short-term intraocular pressure elevation and a small amount of intraocular hemorrhage were more common. Therefore, we believe that the combination of vitreous surgery and tumor resection is a safe treatment.

In the present study, there were no statistical differences between the two groups, except gender before surgery. Compared with the baseline level, the improvement of BCVA at 6 months and the last follow-up visit were more pronounced in Group R than in Group C. And there were more eyes with BCVA improved more than 0.2 LogMAR. In the two groups, there were also some patients with decreased visual acuity compared with the baseline level, but the reasons were quite different. The loss of visual acuity in Group C was mainly related to the macular disease secondary to the tumor noninactivation, whereas the vision reduction in Group R was mainly due to the lens opacity. Hence, we believe that the combination of vitreous surgery and tumor resection is an effective treatment.

## 5. Conclusions

We found that RVPT may be more likely to occur in nonhigh myopia eyes. Epimacular membrane and vitreous hemorrhage are common causes of vitreous surgery in RVPT patients. However, those with inflammatory opacities in the vitreous should be fully pupil dilated to check the peripheral fundus and be alert to the possibility of RVPT. Vitreous surgery is an effective method to treat RVPT-related complications. Compared with conservative treatment during surgery, the surgical removal of the tumor did not increase the risk of surgical complications. It completely inactivated the tumor, which was more conducive to the vision recovery and prevented long-term complications associated to the noninactivated tumors. It is a safe and effective treatment.

## Figures and Tables

**Table 1:** Summary of RVPT vitrectomy procedures.

	Article				Patient			Tumors		First treatment		Second treatment		Third treatment	
	Author	Year	Country	Journal	Eyes	Age	BCVA pre (post)	Type	EndoR	Tumor	Vitrect	Tumor	Vitrect	Tumor	Vitrect
1	Castro-N [17]	2016	USA	OmanJ Ophthalmol	1	29	0.05 (0.4)	P	no	Cryo	no	no	SEMM		
2	Garcia [6]	2015	Spain	Ophthalmic Research	31	37.8 (12.7–65.6)	0.46 (?)	P/S = 23/8 (PP × 5; RP × 2; Tp × 1)	no	17 (Ph + Cryo × 6; Ph × 17; Cryo × 8)	17 (EMM × 10; RRD × 2; VH × 2; TRD × 1; SRD × 1; SHH × 1)	11 (Ph + Cryo × 2; Ph × 6; Cryo × 1; Ru × 1; TTT × 1)	8 (EMM × 5; RRD × 1; MH × 1;VH × 1)	3 (Ph × 2 + 1)	2 (EMM × 2)
3	Nakamura [18]	2013	Japan	Retinal Cases Brief Reports	1	78	0.5 (0.03)	Unclear	yes	no	RT + NVG				
4	Saito [19]	2013	Japan	Retina	1	54	0.5 (1.2)	S (Coats)	yes	IVB	RT + RRD		RRRD		
5	Makdoumi [8]	2011	Sweden	Acta Ophthalmol	8	36	0.0 6 (0.1)	S (Asteroid hyalosis	no	Cryo + Ph	EMM	Cryo	CE		
						66	0.3 (0.3)	P	no	Cryo			EMM + CE		
						74	0.1 (0.2)	P	no	Cryo		Ph	EMM + CE		
						14	0.6 (1.0)	P	no	Ph	EMM				
						14	0.3 (0.6)	P	no	Cryo + Ph	EMM				
						60	0.1 (0.1)	S (chorioretinitis)	no	Cryo	EMM	Tr			
						65	0.2 (0.4)	S (uveitis)	no	Cryo	EMM				
						62	0.4 (0.5)	P	no	Ph × 5		Cryo	EMM		
6	Steven [20]	2010	USA	Arch Ophthalmol	1	31	0.4 (0.5)	Unclear	yes	no	RT + VH				
7	Gibran [16]	2008	USA	Clin Exp Ophthalmol	3	20	CF (0.3)	P	yes	Cryo + IVB + PDT			RT + EMM + CE		SO removal
						33	0.1 (0.2)	P	yes	Cryo + IVB			RT + EMM + CE		IOL-e+(sfau)
						27	CF (0.15)	S (uveitis)	yes	Cryo + IVB			RT + CE		IOL-e+(sfau)
8	Youssef [14]	2007	USA	Reinal Cases Brief Reports	1	39	0.05 (0.8)	Unclear	no	Cryo			SME		
9	Shankar [21]	2007	UK	Eye	1	34	0.25 (unclear)	P	no	Cryo	no	Cryo	EMM		
10	Liang [22]	2007	China	Ophthalmology	1	17	0.6 (0.9)	P	yes	no	RT				

CE = cataract extraction; Cryo = cryotherapy; EMM = epimacular membrane; EndoR = endoresection; IOL = intraocular lens; IOL-E+ = IOL explanation + SO removal + artisan IOL insertion; MH = macular hole; NVG = neovascular glaucoma; IVB = intravitreal bevacizumab; PDT = photodynamic therapy; PP = pars planitis: Ph = photocoagulation; RP = retinitis pigmentosa; Ru = ruthenium plaque; RRD = rhegmatogenous retinal detachment; RRRD = recurrent RRD; RT = resected tumor; SFAU = severe fibrinous anterior uveitis; SRD = serous retinal detachment; SHH = subhyaloid hemorrhage; SME = submacular exudates: SO = silicon oil; Tp = toxoplasmosis; TRD = tractional retinal detachment; TTT = transpupillary thermotherapy; Tr = triamcinolon; VH = vitreous hemorrhage; Vitrect = vitrectomy.

**Table 2:** Baseline characteristics of patients.

Case	Sex/Age/Eye	SD	RT	Symptom	DS (months)	BCVA (logMAR)			FT (months)	AL (mm)	RE (D)	IOP (mmHg)	FD	Tumors					
						Pre-op	Post-op 6m	Last						Ty.	Pd	B	N	L	S (PD)
1	F/55/R	—	no	DV	36	0.7	0.15	0.2	19	21.61	—	16.6	EMM	P	no	no	1	IT	2
2	F/42/R	Ht	no	Fr; M	8	0.3	0.3	0.2	8	22.5	−0.25	14.0	EMM	P	no	no	1	IT	4
3	F/42/L	PT	no	DV	12	0.1	0.05	0	38	23.92	0.25	16.4	EMM; TRD	P	no	no	1	IN	2
4	F/58/R	CE	no	DV; RE	8	2	1.7	1.3	31	23.14	5.00	10.3	EMM; Uv; REx; SRF	S	yes	no	1	T	2
5	M/65/R	—	no	DV	1	2.6	0	0	—	22.99	—	7.9	VH (4)	P	no	no	1	IT	0.5
6.1	F/42/L	Ht	no	DV; Fr	0.25	0.3	1.3	1.3	8	23.32	0.00	13.9	Uv; REx; SRF	S	yes	no	1	IT	2
7.1	F/64/R	Ht	no	DV; Fr; FL	6	0.7	1	1	6	22.84	0.50	16.3	TRD: EMM	P	no	no	1	ST	6
7.2	F/64/L	Ht	no	DV	3	0.15	0.1	0.1	6	22.52	0.00	20.8	EMM	P	yes	no	1	T	2
8	F/60/L	—	no	DV	36	0.2	0.1	0.1	17	23.87	0.25	12.3	MH; EMME: TRD	P	yes	no	1	IT	4
9	F/43/R	—	no	Fr	0.5	0	0	0	6	23.57	0.00	11.7	VH (1)	P	yes	no	1	T	2
10	M/47/L	AS	yes	DV	12	1.7	0.1	0.1	23	23.5	−1.00	10.9	VH (3); EEM	P	yes	no	1	IT	2
11	M/69/R	Ht	yes	DV	2	2.3	0.4	0.5	5	23.92	1.50	13.4	VH (4)	P	no	no	1	IN	—
12	F/65/L	—	yes	DV; Fr	1	0.05	0.1	0.2	6	22.61	−0.25	14.9	VH (1)	P	yes	yes	1	T	3
13	F/41/L	—	yes	DV; Fr	4	1	0.4	0.7	15	22.91	−3.25	10.1	VH (1); EEM; SRF; Uv; Rex	S	yes	yes	1	IT	2.5
14	M/37/L	—	yes	DV; M	1	0.5	0.1	0.1	6	24.67	−1.75	12.4	EMM; Uv	P	yes	no	1	ST	1.5
15	F/34/L	—	yes	DV; M	2	1.85	0.4	0.4	6	23.55	0.00	19.4	VH (1); EEM; Uv; TRD	S	yes	yes	1	IN	2
16	M/25/L	—	yes	DV; Fr	4	0.8	0.1	0.4	39	24.05	−0.25	12.4	EMM; Uv; SRF;	S	yes	no	1	T	3
6.2	F/42/L	Ht	yes	DV; VFD	5	1.3	1	1	30	23.32	0.00	17.3	Uv; REx; SRF	S	yes	no	1	IT	2

F, female; M, male; SD, systemic disease; RT, resection of tumor; DS, duration of symptoms; BCVA, best-corrected visual acuity; Pre-op, preoperative; Post-op 6m, postoperative 6 months; last, last visit; FT, follow-up time; AL, axial length; RE, refractive error; IOP, intraocular pressure; FD, fundus disease; Ty., type; Pd, preoperative diagnosis; B, B ultrasound; N, number; L, location; S, size; PD, papillary diameter; Ht, hypertension; PT, pulmonary tuberculosis; CE, cancer of endometrium; AS, ankylosing spondylitis; Fr, floater; DV, decreased vision; VFD, visual field defect; FL, flash of light; M, metamorphopsia; RE, red eye; EMM, epimacular membrane; VH(degree), vitreous hemorrhage; Uv, uveitis; SRF, subretinal fluid; TRD, tractional retinal detachment; RRD, rhegmatogenous retinal detachment; Rex, retinal exudation; MH, macular hole; IT, infratemporal; IN, infranasal; T, temporal; ST, superotemporal.

**Table 3:** Comparison of the qualitative features between the two groups.

	Group C	Group R	*t*	*P*
Age	53.5 ± 10.14	45 ± 15.07	1.429	0.172
Duration of symptom (months)	11.08 ± 13.68	3.88 ± 3.6	1.597	0.14
Follow-up time (months)	15.44 ± 11.96	16.25 ± 13.07	−0.133	0.896
Axial length (mm)	23.03 ± 0.75	23.57 ± 0.65	−1.558	0.14
Spherical equivalent (D)	0.64 ± 1.65	−0.63 ± 1.41	1.687	0.112
Intraocular pressure (mmHg)	14.7 ± 3.19	13.85 ± 3.18	0.549	0.591
BCVA (logMAR)				
Pre-op	0.49 ± 0.62	1.19 ± 0.75	−2.097	0.053
Post-op 6 months	0.52 ± 0.64	0.33 ± 0.31	0.824	0.426
Last	0.47 ± 0.56	0.43 ± 0.31	0.192	0.851
Post-op 6m vs pre-op	0.03 ± 0.43	−0.86 ± 0.70	3.201	0.006^∗^
Last vs post-op 6m	−0.06 ± 0.14	0.10 ± 0.13	−2.398	0.03^∗^
Last vs pre-op	−0.03 ± 0.48	−0.76 ± 0.73	2.407	0.033^∗^

**Table 4:** Comparison of the quantitative results between the two groups.

	Group C	Group R	*X*	*P*
Sex (male/female)	0/9	4/4	Fisher	0.029
Eye (left/right)	4/6	7/1	Fisher	1.429
Tumor activity (no/yes)	6/3	8/0	Fisher	0.008

## Data Availability

The data used to support the findings of this study are available from the corresponding author upon request.
